# Revealing the role of organic cations in hybrid halide perovskite CH_3_NH_3_PbI_3_

**DOI:** 10.1038/ncomms8026

**Published:** 2015-04-27

**Authors:** Carlo Motta, Fedwa El-Mellouhi, Sabre Kais, Nouar Tabet, Fahhad Alharbi, Stefano Sanvito

**Affiliations:** 1 School of Physics, AMBER and CRANN Institute, Trinity College, Dublin Dublin 2, Ireland; 2 Qatar Environment and Energy Research Institute, PO Box 5825, Doha, Qatar; 3 Department of Chemistry and Physics, Purdue University, West Lafayette, Indiana 46323, USA

## Abstract

The hybrid halide perovskite CH_3_NH_3_PbI_3_ has enabled solar cells to reach an efficiency of about 20%, demonstrating a pace for improvements with no precedents in the solar energy arena. Despite such explosive progress, the microscopic origin behind the success of such material is still debated, with the role played by the organic cations in the light-harvesting process remaining unclear. Here van der Waals-corrected density functional theory calculations reveal that the orientation of the organic molecules plays a fundamental role in determining the material electronic properties. For instance, if CH_3_NH_3_ orients along a (011)-like direction, the PbI_6_ octahedral cage will distort and the bandgap will become indirect. Our results suggest that molecular rotations, with the consequent dynamical change of the band structure, might be at the origin of the slow carrier recombination and the superior conversion efficiency of CH_3_NH_3_PbI_3_.

## Abstract (web summary)

Hybrid halide perovskite solar cells are studied as easy to fabricate compounds for efficient solar cell devices. Here, the authors use density functional theory calculations to show that the orientation of the organic molecules in these materials is crucial for their electronic and solar conversion properties.

## Graphical abstract

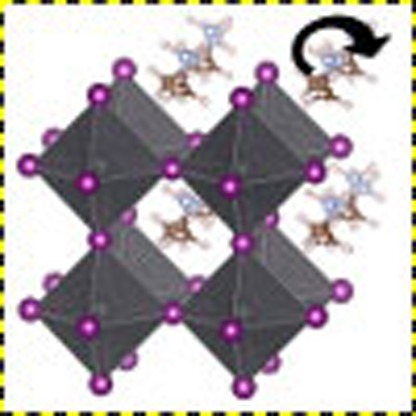

Very few materials have taken a research field by storm as the hybrid halide perovskites have done in the last 2 years in the solar cell community^1^. These are compounds with the standard AMX_3_ perovskite structure, where X is the halide iodine, M is lead, while the remaining cation position, A, is taken by an organic molecule, in this case methylammonium (MA), CH_3_NH_3_. The success of such compounds is that high-efficiency solar cells can be fabricated cheaply from the liquid phase^2^,^3^ and that the efficiency can be tuned by controlling their structural order and composition^4^,^5^. Particularly intriguing is the fact that high efficiencies are achieved even for planar cells^6^, indicating that the charge separation can occur in the hybrid perovskite absorber and that efficient charge diffusion takes place for both electrons and holes^7^.

Part of this behaviour can be related to the unusual situation concerning possible intrinsic defects. Recent electronic structure calculations^8^,^9^ have shown that the dominant simple or complex defects in CH_3_NH_3_PbI_3_ produce only shallow defect levels, while those able to act as deep traps have a high formation energy. Such observation suggests that, under ideal conditions, deep-level point defects cannot act as non-radiative recombination sites—a fact that can partially explain the relative long exciton lifetimes and diffusion lengths observed in these materials. At the same time, the absence of deep levels may explain also the large open-circuit voltage measured. However, despite all these hints, many fundamental questions remain open. In particular, it is still not clear what is the role played by the organic molecules in the efficiency of these compounds. Theoretical investigations have now built a consensus that the MA group does not have any significant contribution to the electronic structure around the band edges^8^,^10^,^11^ and suggest that the only role of the molecules is to stabilize electrostatically the perovskite structure.

Interestingly, not even the geometrical arrangement of the MA group is known in detail. At low temperature (below 150 K), the crystal adopts an orthorhombic (*Pnma*) structure, in which the PbI_6_ octahedra are strongly deformed assuming a rectangular basal plane. Such deformation restricts the rotational degrees of freedom of MA in the rhombus-shaped interstitial region, thus imposing a spatial ordering to CH_3_NH_3_. In this case, the organic cation is pinned and can only rotate along the C–N axis. As the temperature is increased and the material progressively assumes a cubic structure (*Pm-3m*), by passing through a tetragonal one (*I4/m*), the CH_3_NH_3_ molecules become free to rotate between the octahedral cages. Above room temperature, such rotation is fast, to a point where both crystallographic analysis^3^,^12^ and NMR measurements^13^ have shown that the exact location of the MA groups cannot be determined.

Here we conduct density functional theory (DFT) calculations for CH_3_NH_3_PbI_3_ in the cubic phase, by taking into consideration the experimentally reported evidence of the fast rotation of CH_3_NH_3_. This means that we do not limit our analysis to CH_3_NH_3_ oriented along the (100) or (111) direction, for which a higher symmetry is maintained, but we also explore cases where the symmetry is lowered. We find that such symmetry lowering has profound consequences on the electronic structure, namely that the bandgap changes from direct to indirect. Crucially, such symmetry-lowering configurations represent local minima in the free energy surface of the crystal and they are stabilized by van der Waals (vdW) interactions. These are the key ingredients not only for obtaining accurate lattice parameters^12^,^14^,^15^,^16^,^17^ (see Supplementary Fig. 1) but also for the internal geometry. Our calculations then return a picture of the CH_3_NH_3_PbI_3_ as a ‘dynamical' bandgap semiconductor, in which the exact position of the conduction band minimum depends on the particular spatial arrangement of the molecules. Importantly, our results are robust against bandgap corrections and spin–orbit interaction^11^,^18^,^19^,^20^,^21^, and deliver an absorption spectrum in good agreement with experimental data near the absorbtion edge^7^,^22^.

## Results

### Geometrical optimization

Let us start the discussion by presenting the relaxed crystal geometries. Initially, all the calculations have been performed at the level of the generalized gradient approximation (GGA)^23^ to the DFT exchange and correlation functional, including vdW interactions^24^ (see computational details section) and without explicitly considering the spin–orbit coupling (SOC). Additional calculations beyond the GGA and including SOC will be discussed later in relation to the electronic structure.

**Figure 1:**
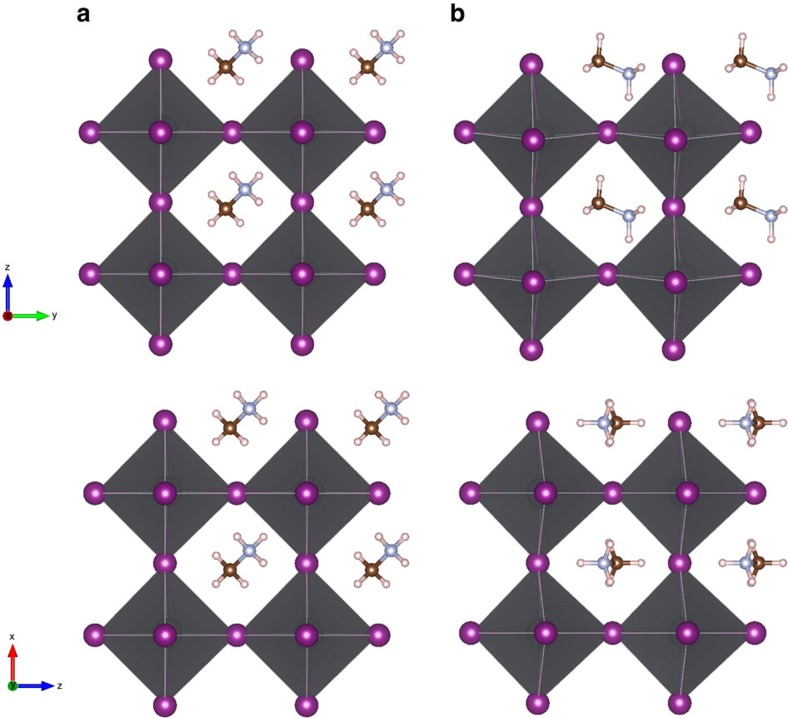
Relaxed structures of the cubic phase of CH_3_NH_3_PbI_3_. Relaxed structures of the cubic phase of CH_3_NH_3_PbI_3_ for two different orientations of the cations, namely along (**a**) (111) and (**b**) (011). (**b**) The views along the *xy* and *xz* planes are shown in the upper and lower panels, respectively.

In Fig. 1, the relaxed structures for the cubic phase of CH_3_NH_3_PbI_3_ are presented along the *xy* and *xz* planes for two different orientations of the CH_3_NH_3_ cation. Given the periodic boundary conditions, these structures effectively represent the specific case of an infinite array of perfectly aligned molecular dipoles. The relaxation process is extremely sensitive to the initial conditions. When we start the geometrical optimization with the molecular cation oriented along the (111) direction, the structure relaxes maintaining the same orientation (Fig. 1a). In contrast, by starting from a (001)-oriented molecule, the relaxation may end up with CH_3_NH_3_ along (011) (Fig. 1b) or an equivalent direction. However, it is important to point out that the cation energy landscape is very shallow. Indeed, both configurations represent local energy minima, with an energy difference of only about 20 meV in favour of (011). In both cases, the lattice parameter is calculated in excellent agreement with the experimental value of 6.329 Å (ref. 25) and an error of <0.8% (see Supplementary Table 1 and Supplementary Fig. 1). If one does not include vdW forces in the relaxation, the discrepancy with the nominal value will become >2%, that is, the crystal structure is not properly described. Furthermore, the angle formed by the molecular axis and the (001) direction increases from 16 to 25° on inclusion of dispersion forces. To make a well-grounded comparison with previous calculations^11^,^26^, reporting no effect of MA on the structure, we started the relaxation from the X-ray diffraction solved structure with CH_3_NH_3_ along (100)^27^, whose band structure is presented in Supplementary Fig. 2.

By inspecting the relaxed structures, a prominent difference between the (111) and the other two geometries appears. In fact, in the case of (011), the rotation of the cation induces a deformation and a symmetry reduction of the inorganic PbI_6_ octahedra. The result of such PbI_6_ octahedra distortion is that the Pb–I bonds do not lie parallel to the crystal directions, but instead they slightly deviate by ∼6°. Unlike the (111) case, the unit cell becomes pseudocubic with the lattice parameters along the *y* and *z* directions being 1% larger than that along *x*. Hence, it is clear that the dispersive forces are critical for the internal geometry optimization and consequently, as we will see next, for the electronic behaviour of hybrid perovskites.

### Electronic properties

**Figure 2:**
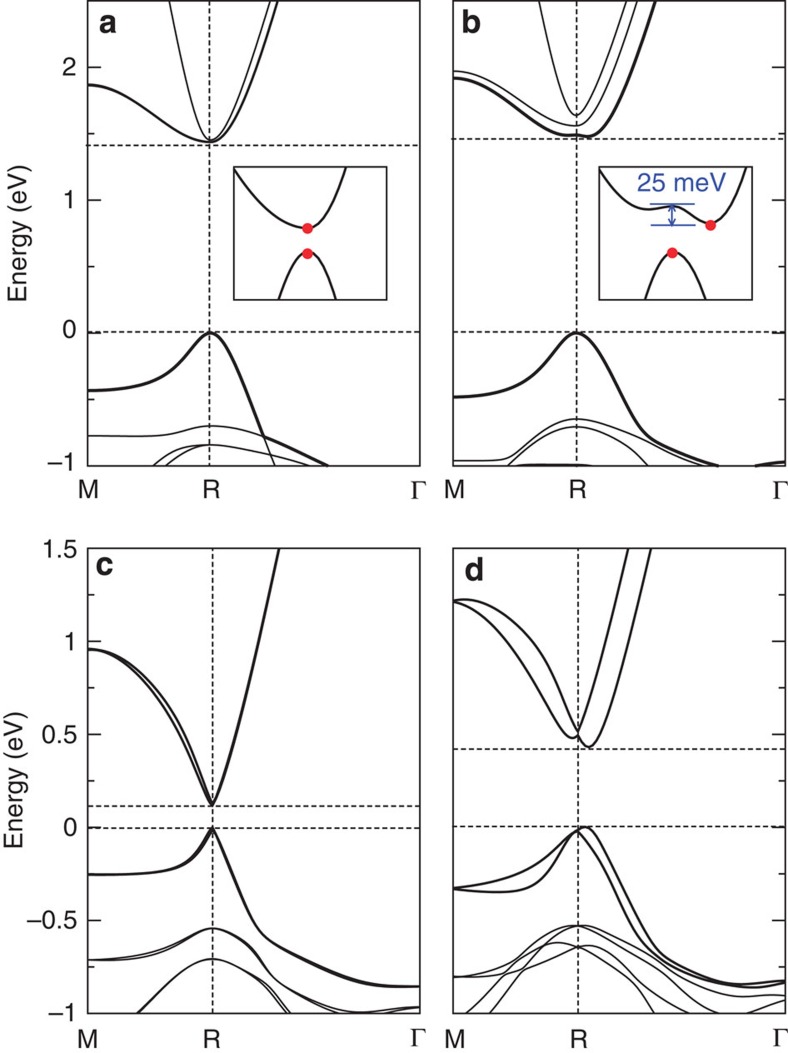
Band structure of the fully relaxed CH_3_NH_3_PbI_3_ crystal. The bands are shown for molecule orientations along (**a**) (111) and (**b**) (011) direction. The insets show a magnification of the bands (which have been shifted in energy for convenience) around the bandgap and highlight the changes in the VBM and CBM caused by the rotation of CH_3_NH_3_. Note that for the (011) orientation the bandgap becomes indirect. The relativistic SOC band structures are shown in panels (**c**) and (**d**) for (111) and (011).

**Figure 3:**
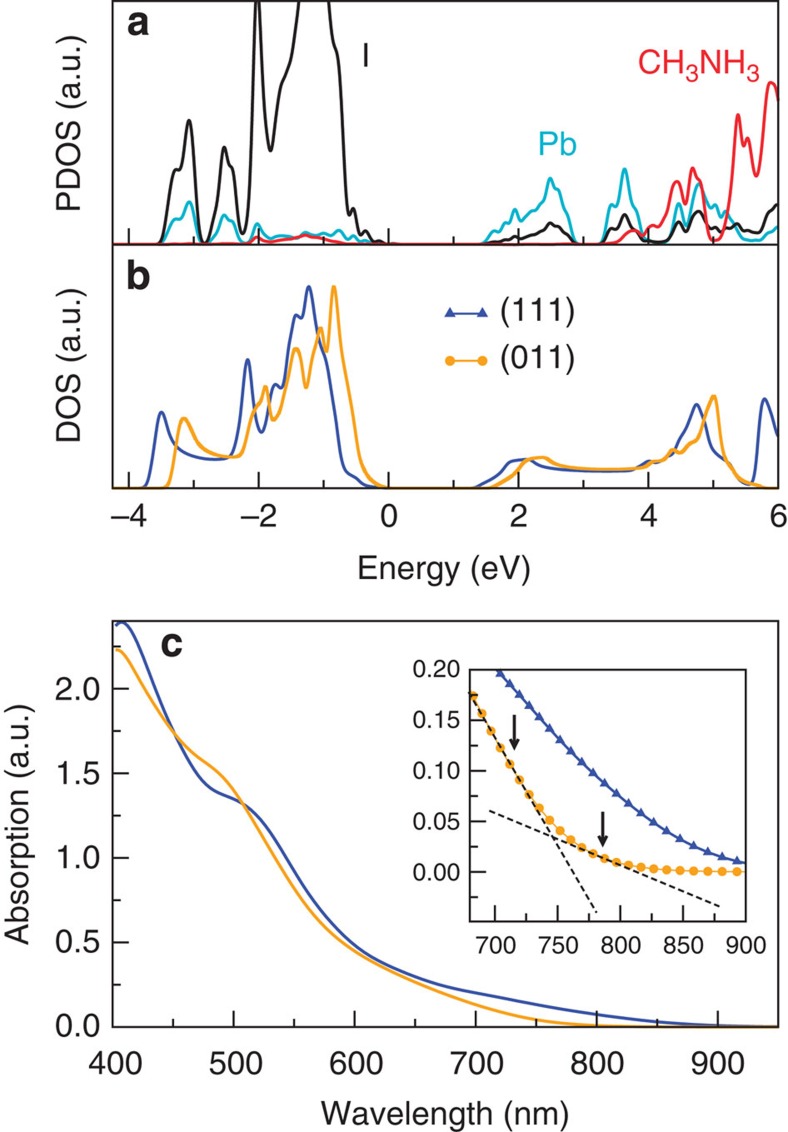
Electronic and optical properties of the cubic phase CH_3_NH_3_PBI_3_. (**a**) Projected density of states (DOS) for the case of the (111)-oriented molecule on the Pb (cyan), I (black) species and the CH_3_NH_3_ (red). (**b**) DOS as a function of the chemical potential for the molecule oriented along the (111) and (011) directions are displayed. (**c**) The calculated absorption spectra are plotted for the different orientation of CH_3_NH_3_ clearly showing the impact the organic cation rotation. The inset in **c** is a magnification of the same quantity around the low-energy region.

The fine differences in the crystal geometries have a qualitatively strong impact on the electronic structure. In Fig. 2, we show the electronic band structure along some high-symmetry points of the Brillouin zone calculated with and without including fully relativistic SOC. For both orientations, the energy gap computed without SOC lies in the 1.5–1.7 eV range, close to the experimental value of about 1.55 eV (refs 22, 28). However, as already shown^5^, this agreement is due to a fortuitous cancellation of errors, namely the GGA underestimates the bandgap, but this is counterbalanced by the lack of the spin–orbit interaction. It is well known that the bottom of the conduction band mainly originates from the *p* orbitals of Pb, while the top of the valence band is derived from the *p* orbitals of I. The highest occupied molecular orbital of MA is found deep below the valence band, ∼5 eV below the valence band maximum (VBM). Thus, one may argue that CH_3_NH_3_ does not play any role in the optical and electronic response of such materials, but that rather it does only contribute to their structural cohesion. However, a careful analysis of the density of states projected on the various atoms (see Fig. 3a and Supplementary Fig. 3) reveals that there is a small contribution ∼0.5 eV below the VBM attributable to the organic molecules. This indicates that indeed there is an interaction between CH_3_NH_3_ and the inorganic PbI_6_ octahedra, in the form of hydrogen bonds between N and I.

A closer inspection to the band structures reveals that the orientation of CH_3_NH_3_ has a profound impact on the nature of the bandgap. In fact, while for the (111) case the gap is direct at the R point, the cationic rotation induces a transition to an indirect gap, and when MA is along (011) the conduction band mininum (CBM) shifts along the R→Γ line. Accordingly, the vertical energy difference between the CBM and the conduction band energy at R is 25 meV, as shown in the inset of Fig. 2b. The effect persists when SOC is turned on, as shown in Fig. 2c,d. In this case, a slight shift of the VBM towards Γ occurs as well, reducing the CB vertical energy difference approximately to half the non-SOC value. We stress here that the (011) orientation is not a special case—the indirect bandgap does appear for all the equivalent orientations, namely (0±11), (01±1), (±101), (±110), (10±1) and (01±1). As an example, in Supplementary Fig. 4, we show the (0–11) case, for which the CBM shifts in the direction opposite to that shown in Fig. 2, namely R→M.

Such a gap transformation is the most remarkable result of the interaction between the organic molecules and the inorganic framework, which is driven by the dispersive forces. As a result, CH_3_NH_3_ exerts strain on the PbI_6_ octahedra and changes the nature of the bandgap. This is unusual in the perovskite world and it is a unique peculiarity of such hybrid systems. Typical inorganic perovskites such as SrTiO_3_ respond to very small uniaxial strains, as those induced by the antiferrodistortive phase transition, by splitting their threefold degenerate CBM^29^. To the best of our knowledge, very few semiconductors see their bandgap changing from direct to indirect under the effect of strain. An example is given by layered transition metal chalcogenides, like for instance, MoSe_2_ single layer, where the gap change is attributed to phonon softening^30^. Intriguingly, also for CH_3_NH_3_PbI_3_, we observe some softening of our calculated low-energy optical phonon modes, when the MA molecule rotates along the (011) directions. In particular, the calculated energy red shift is of the order of 10 cm^−1^ (see Supplementary Fig. 5).

We have carefully verified that the indirect bandgap is not an artifact of our computation method. First, we analysed the relaxation in a 2 × 2 × 2 (96 atoms) supercell to establish whether further low-symmetry configurations are possible. The supercell converged geometries are very similar to the unit cell geometries repeated in space due to periodic boundary conditions. The calculations reveal no particular changes, although the size of the supercell would allow the PbI_6_ octahedra to rotate or tilt. Consequently, the features of the bandgaps are preserved both at the qualitative as well as the quantitative level.

Second, we have tested different vdW functionals by comparing the geometries obtained with the Tkatchenko–Scheffler (TS) scheme^24^ to those calculated with the empirical Grimme's method^31^ and with a recently developed sophisticated many-body dispersion correction^32^. As shown in Supplementary Fig. 1, the unit cell volume compares well with the experimental value for all the vdW-corrected functionals, although that calculated with the TS scheme is closer. Thus, in general, the TS and Grimme's pairwise dispersion methods provide a sufficiently good description of our system as found in a recent work on molecular crystals^17^. In any case, the indirect bandgap is found for all the calculated geometries, including those obtained with the many-body dispersion correction. In contrast, when the dispersion interactions are not included, the gap remains direct since the structure does not present enough distortion. Then, we have performed calculations using the HSE06 (ref. 33) screened hybrid functional by starting from the relaxed structures. Also, in this case, the bandgap remains indirect (see Supplementary Fig. 3), although the overall energy distance between the CBM and the VBM increases, as expected.

Finally, we have performed additional calculations to establish whether or not the observed effect is the result of the interaction of the MA dipole with its periodic images. We have then verified that the position of the VBM and the CBM does not shift (i) on flipping the molecule's dipole moment and (ii) on applying a rigid rotation of the molecule from the 111 to the 110 orientation (Supplementary Fig. 6). This confirms that it is the distortion of the inorganic PbI_6_ octahedra to govern the shape of the CBM and VBM and not the dipole moment of the organic molecule.

As a last element of our analysis, we have investigated the relative stability of the different molecular orientations. In particular, we have performed nudged elastic band calculations and evaluated the barrier height for an MA rotation from (011) to (111), taking into account the possible changes in the cell geometry and the lattice vectors. We have found a barrier of 20 meV (1.929 kJ mol^−1^), which is less than *k*_B_*T*, and it is similar to values found recently in literature^34^. This confirms the concept that above room temperature the molecules continuously rotate. Given the strong dependence of the position in *k*-space of the CBM and the VBM on the cell geometry, the fast molecular rotation implies that the nature and shape of the bandgap are rapidly-varying functions of time. Recently, Mosconi *et al.*^35^ published Car–Parinello molecular dynamics simulations for a 2 × 2 × 2 CH_3_NH_3_PBI_3_ supercell at 319 K, showing fluctuations of ±0.1–0.2 eV in the position of the VBM, and thus giving evidence that the size of the bandgap changes as function of the MA rotation. Here we go a step forward and demonstrate that for some molecular orientations the bandgap turns from direct to indirect.

### Optical absorption

To seek further evidence of the presence of the indirect bandgap, we have calculated the perovskite's optical absorption and compared it with available experimental data. The calculations have been performed over the electronic structure obtained with the GGA including vdW interaction in the independent particle approximation and without SOC. Although the fortuitous cancellation of errors returns a bandgap close to experiments, we concentrate here on the shape of the spectrum around the absorption edge. The calculations are performed for the two orientations of the CH_3_NH_3_ molecule, and the spectra are presented in Fig. 3c.

When MA is aligned along the (111) direction, the bandgap is direct and measures 1.423 eV=871 nm. The absorption profile then increases monotonically from that value, indicating the possibility of direct transitions across the band edges. In contrast, when CH_3_NH_3_ is placed along (011) the bandgap is indirect, and in general slightly larger than that corresponding to the (111) orientation. In particular, for (011), we find a direct gap of 1.611 eV=769 nm and an indirect one of 1.629 eV=761 nm. Here the shape of the absorption spectrum is quite different from the (111) case. We note a ‘two-step' absorption profile (see the inset of Fig. 3c), with a small absorption amplitude developing at the band edge followed by a significantly more pronounced increase at around 750 nm. This reflects the indirect nature of the bandgap and the fact that indirect transitions are not allowed, unless assisted by phonons. Certainly, the level of theory used here is not complete and one should use the many-body methods for deriving a more quantitative picture^36^; however, the inclusion of excitonic effects is not necessary to make our main point about the shape of the spectrum around the absorption edge. In fact, the differences between the direct and indirect bandgap are very evident from the inset of Fig. 3c, and should be compared with the typical experimental absorption spectrum measured at 300 K (ref. 4). This latter features a small amplitude around the bandgap followed by a rapid increase after about 50 nm, which seems to be a robust feature in the experimentally measured spectra, regardless of the morphology of the material^7^,^22^,^37^ and resistent to film quality degradation^38^.

Whether or not such a near-edge feature can be attributed to the indirect bandgap is however difficult to establish with certainty. In fact, at 300 K, the MAs are free to rotate and sample the many quasi-equivalent (011) and (111) orientations. Thus, it is expected that no sharp indirect bandgap can be observed, but rather a smooth absorption spectra starting around the average bandgap value. Interestingly, we point out that our finding could help explaining the recently observed line broadening in photoluminescence experiments^39^, attributed to coupling with phonons. In fact, low-energy phonons are associated with cation rotations, which in turn would shift the CBM to different positions. As a result, the range of optical gap would change as a function of temperature, thus broadening the emission spectrum. Measurement of the optical absorption and thermal characteristics using very high-sensitivity techniques such as photothermal deflection spectroscopy might reveal the existence of the indirect bandgap by fine lowering of temperature <300 K thus freezing the MA (011)-like orientations.

## Discussion

Absorbing light efficiently and transporting the photocurrent with some gained voltage are the most important aspects in the design of a solar cell. The two processes can be characterized, respectively, by an empirical absorption length, *L*_*α*_, inversely proportional to the absorption^40^,^41^, and by the carrier diffusion length, *L*_diff_. This is a measure of the mean distance travelled by carriers before recombining^42^,^43^,^44^; it is directly proportional to the carrier lifetime, *τ*, and it depends on several factors (mobility, carrier concentration and so on). In solar cells, it is essential to fulfil the condition *r*=*L*_diff_/*L*_*α*_>1 to ensure that the photogenerated carriers can be extracted and collected. For practical reasons, *r*≫1 is required for high power conversion efficiency^40^,^41^,^42^,^45^.

In general, direct bandgap semiconductors, such as GaAs, have strong absorption and thus short *L*_*α*_, but this comes with fast carrier recombination and hence relatively small *L*_diff_. The situation is opposite when the bandgap in indirect (for example, in Si). An ideal balance between *L*_*α*_ and *L*_diff_ is present in some indirect bandgap semiconductors such as Cu_3_N (refs 41, 42), where the direct transition starts a few *k*_B_*T* above the indirect gap, yielding a short *L*_*α*_. The small difference between the indirect gap and the lowest direct transition ensures that the absorption is not greatly affected by the indirect gap till the direct transitions start to dominate the absorption spectrum. In contrast, the lifetime of the photogenerated carriers, which relax to the band edges in the indirect gap semiconductors, is longer than that of the direct ones by orders of magnitudes^43^ ensuring a large *L*_diff_. This class of indirect semiconductors promises remarkable improvement in efficiency via the large *L*_diff_/*L*_*α*_ ratio^41^,^42^.

From this short discussion and our results at hand, we can speculate on the possible origin of the high efficiency in hybrid perovskites solar cells. Our calculations establish that, under certain conditions of molecule alignment, the bandgap of the high-temperature phase of CH_3_NH_3_PBI_3_ can be indirect and not direct. Note that an indirect bandgap in hybrid organic/inorganic perovskite may also arise as a result of Rashba splitting^10^,^46^. This feature originates from the strain exerted on the PbI_6_ octahedra by the organic molecules, when these points mostly towards one of the unit cell faces rather than along the diagonal, which constitute shallow minima in the total energy profile. The indirect bandgap is tens of meV smaller than the direct one and the position of the CBM with respect to the VBM depends on the specific orientation of the molecules. This analysis delivers a picture of CH_3_NH_3_PBI_3_ as a ‘dynamical' bandgap semiconductor, where the term ‘dynamical' refers to the fact that the position in *k*-space of the CBM depends on the molecular alignment, which in turn fluctuates at room temperature. The calculated absorption spectrum near the band edge, characterized by a shallow amplitude at about 800 nm followed by a rapid enhancement within 50 nm, is very similar for (011) and equivalent orientations of the molecules, indicating that it is a feature robust to the molecular rotation and disorder.

The presence of an indirect bandgap, with only a small displacement in *k*-space with respect to the direct point, suggests that the rapid relaxation of the photoexcited electrons to the conduction band minimum is still possible. This process is incoherent, mediated by acoustic phonons and spontaneously breaks the excitons in free carriers. As a second effect of the collapse of the electrons to the indirect conduction band minimum is that the lifetime of the minority carriers is enhanced, so that radiative recombination is partially suppressed. Such suppression is not complete since the experiments reveal luminescence; however, it contributes to prolong the carrier lifetime. In fact, it is interesting to note that experiments report that the luminescence gets suppressed as the material crystallizes^37^ and that in any case it is sensitive to the sample morphology and quality^47^. Considering that the crystallization process leads to the geometries described here, such suppression of the luminescence on ordering supports our argument. To complete the picture, it is also worth reminding that the absence of strong scattering centres inhibits incoherent recombination, so that *L*_diff_ remains long^48^.

## Conclusions

Despite the tremendous advances in studying and understanding hybrid perovskites from both the theoretical and the experimental point of views, yet the reason behind the extended carrier lifetime in these materials remains a mystery. Our work suggests that the extended carrier lifetimes in CH_3_NH_3_PBI_3_ might be a consequence of the ‘dynamical' position of the conduction band minimum forming a basin around a high-symmetry point in the *k*-space. This proposed dynamical bandgap, induced by the molecular motion, prohibits carrier recombination and favours exciton separation, thus increasing the conversion efficiency of the photvoltaic absorber CH_3_NH_3_PBI_3_. Crucially, all such phenomenology appears to be initiated by the geometry assumed by the molecules following full geometry relaxation. A set of differently oriented CH_3_NH_3_ cation geometries, relaxed without any symmetry constrains using DFT including vdW forces, lead to geometries where the PbI_6_ octahedra distort. The band structure corresponding to such geometries present an indirect bandgap, which persists on bandgap corrections via screened hybrid functionals and SOC. Finally, we propose that a possible way for engineering the efficiency of hybrid perovskites is to induce distortion to the PbI_6_ octahedra either by using strain or organic molecules strongly interacting with the inorganic cages.

## Methods

### DFT calculations

The electronic properties of the hybrid perovskites have been calculated with the all-electron FHI-aims code at the level of GGA in the Perdew–Burke–Ernzerhof^23^ parameterization. Long-range vdW interactions have been taken into account via the TS scheme^24^, which is also constructed over a GGA and a pairwise dispersive potential. The reciprocal space integration was performed over an 8 × 8 × 8 Monkhorst-Pack grid^49^, after performing convergence tests on the total energy and the forces. A preconstructed high-accuracy all-electron basis set of numerical atomic orbitals was employed, as provided by the FHI-aims ‘tight' default option. This is enough to achieve well-converged forces as confirmed by comparing the results with those obtained with the tier2 basis set. The atomic zeroth-order regular approximation was applied to treat relativistic effects. Unconstrained structural optimization was performed with the Broyden–Fletcher–Goldfarb–Shanno algorithm^50^ with with a tolerance of 10^−3^ eV Å^−1^.

Additional calculations, in particular, concerning the use of the hybrid functional HSE06 (ref. 33), were performed using the Gaussian suite^51^, with the periodic boundary condition^52^ code version. The Def2 (ref. 53) series of GAUSSIAN basis sets were optimized following our own procedure, described in ref. 54, for the atomic species of interest in this work. Most numerical settings in Gaussian were left at the default values, for example, uncontrained geometry optimization settings, integral cutoffs and self-consistent convergence thresholds. The standard root mean squared force threshold in Gaussian for geometry optimizations is 450 × 10^−6^ hartrees per Bohr. The default *k*-point mesh of 8 × 8 × 8 was used for the 12 atoms unit cell of CH_3_NH_3_PbI_3_.

Finally calculations involving the spin–orbit interaction have been carried out using the Vienna *ab initio* simulation package (VASP). A plane wave basis set energy cutoff of 520 eV was considered in the calculations and Brillouin zone integration was performed using 8 × 8 × 8 *k*-point mesh centred at the Gamma point. Dispersion correction to the Perdew–Burke–Ernzerhof was done within the method of Tkatchenko and Scheffler (DFT-TS)—recently implemented in the 5.3.5 version of VASP—used to account for vdW interactions. The projector augmented wave pseudopotentials were used for all elements 5*d*^10^6*s*^2^6*p*^2^ valence electron potential was used for the Pb atom. With the same set-up, VASP has been used to compute the band structures with fully relativistic SOC.

The linear optical absorption has been obtained with FHI-aims by computing the momentum matrix elements in a 5-eV energy window around the Fermi level and then by using the Fermi golden rule approximation. A dense *k*-point sampling of 15 × 15 × 15 has been employed to ensure converged spectra.

### Phonon calculations

Phonon spectra were computed with the frozen-phonon approach for a fully relaxed 2 × 2 × 2 supercells using the Phonopy^55^ code in combination with FHI-aims A displacement of 0.001 Å was applied to each atom in the three space directions to compute the dynamical matrix.The phonon DOS used the Γ point sampling, while the full phonon band structure was generated using the Γ, *M*, *X* and *R* high-symmetry points in the Brillouin zone.

## Author contributions

C.M. and F.E.-M. contributed equally to the manuscript. C.M. and F.E.-M. performed the computational research, C.M., F.E.-M., S.K., N.T., F.A. and S.S. discussed the results and contributed to the preparation of the manuscript.

## Additional information

**How to cite this article:** Motta, C. *et al.* Revealing the role of organic cations in hybrid halide perovskite CH_3_NH_3_PbI_3_. *Nat. Commun.* 6:7026 doi: 10.1038/ncomms8026 (2015).

## Supplementary Material

Supplementary Information Supplementary Figures 1-6 and Supplementary Table 1
