# Supplementary material for: Revealing the role of organic cations in hybrid halide perovskite CH3NH3PbI3
Source: Nat Commun. 2015 Apr 27;6:7026. doi: 10.1038/ncomms8026 (PMC4421841; doi:10.1038/ncomms8026)
Supplement: Supplementary Information — Supplementary Figures 1-6 and Supplementary Table 1 [file ncomms8026-s1.pdf]

Supplementary Information

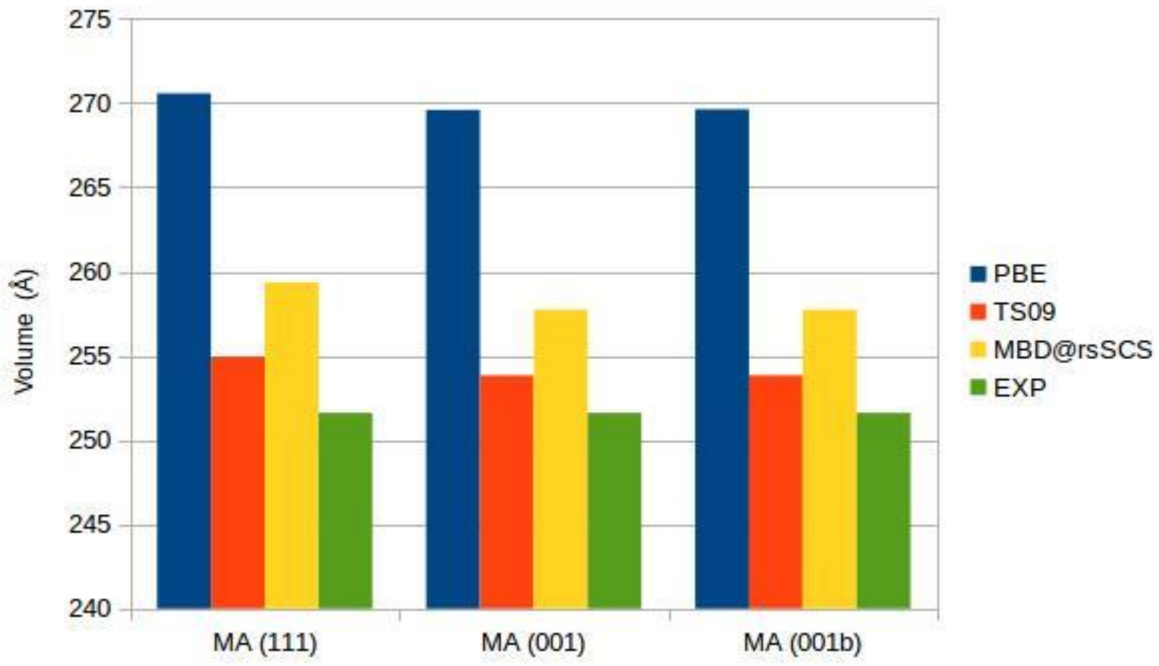

Supplementary Figure 1: After structural optimization of the $CH_3NH_3PbI_3$ unit cell, the resulting relaxed volumes for three distinct orientation of the MA molecules are shown. Volumes were computed with PBE (blue), PBE+TS09-van der Waals interaction (red) and PBE with many-body dispersion interaction correction (MBD) (yellow). The nominal experimental value is also reported (green). The inclusion of van der Waals interactions is crucial for an accurate description of the cell volume. Note that the calculated lattice parameters are athermal, and they are compared with experimental data at room temperature.

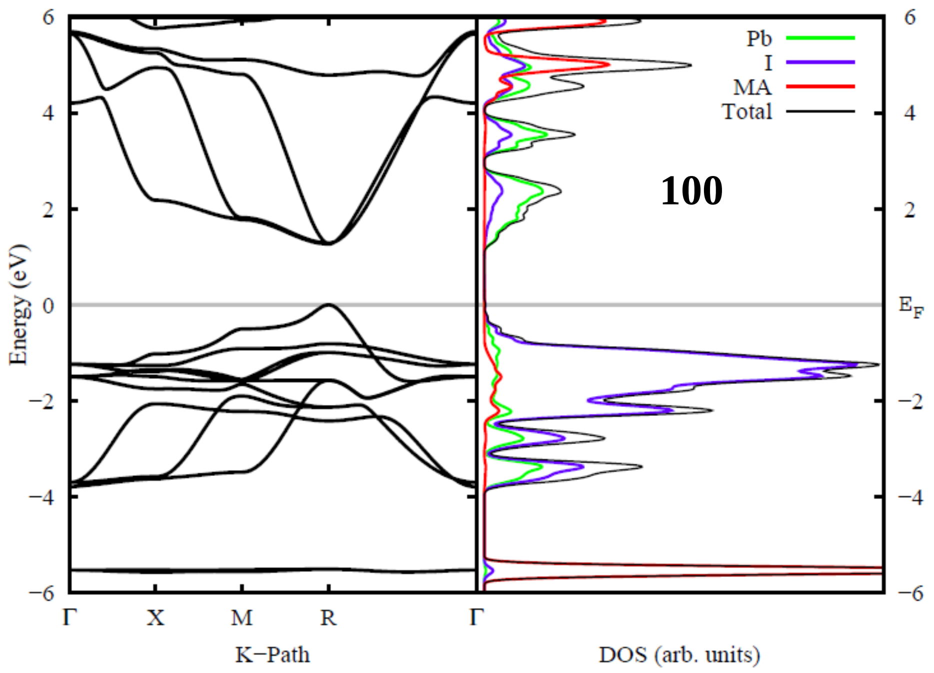

Supplementary Figure 2: GAUSSIAN-calculated electronic band structure and density of states (DOS) of the fully relaxed cubic $CH_3NH_3PbI_3$ unit cell. Results are presented for the PBE functional **without** including van der Waals interactions. The organic site is oriented along (100) direction.

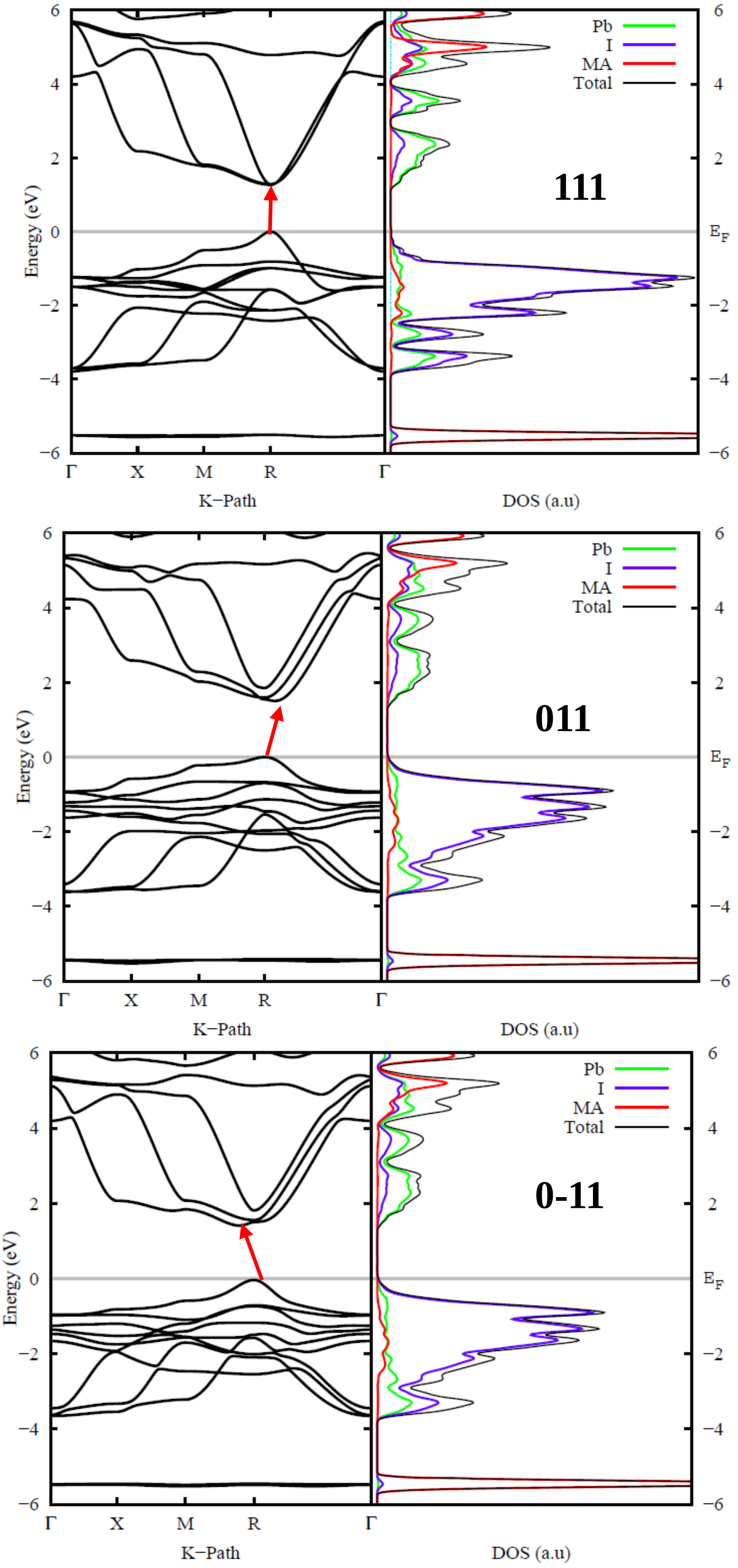

Supplementary Figure 3: GAUSSIAN-calculated electronic band structure and DOS of fully relaxed $CH_3NH_3PbI_3$ along the high-symmetry points computed by using PBE **including** TS09-van der Waals interaction. The CH_3NH_3 organic sites are oriented parallel to the following axis: (111) upper panel (011) middle panel (0-11) lower panel. The red arrows point to the position of the conduction band minimum. This clearly depends on the orientation of the organic molecule.

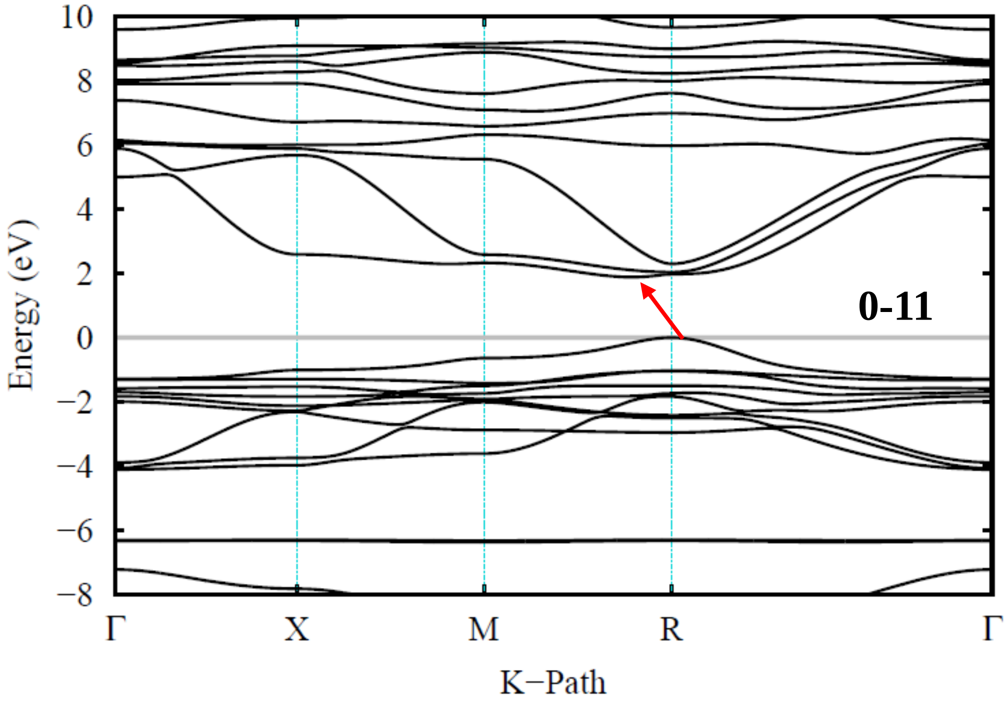

Supplementary Figure 4: GAUSSIAN-calculated electronic band structure of fully relaxed $CH_3NH_3PbI_3$ along the high-symmetry points computed by using the HSE06 hybrid functional, with the organic site oriented along (0-11) direction. An indirect band gap of 1.87 eV is found.

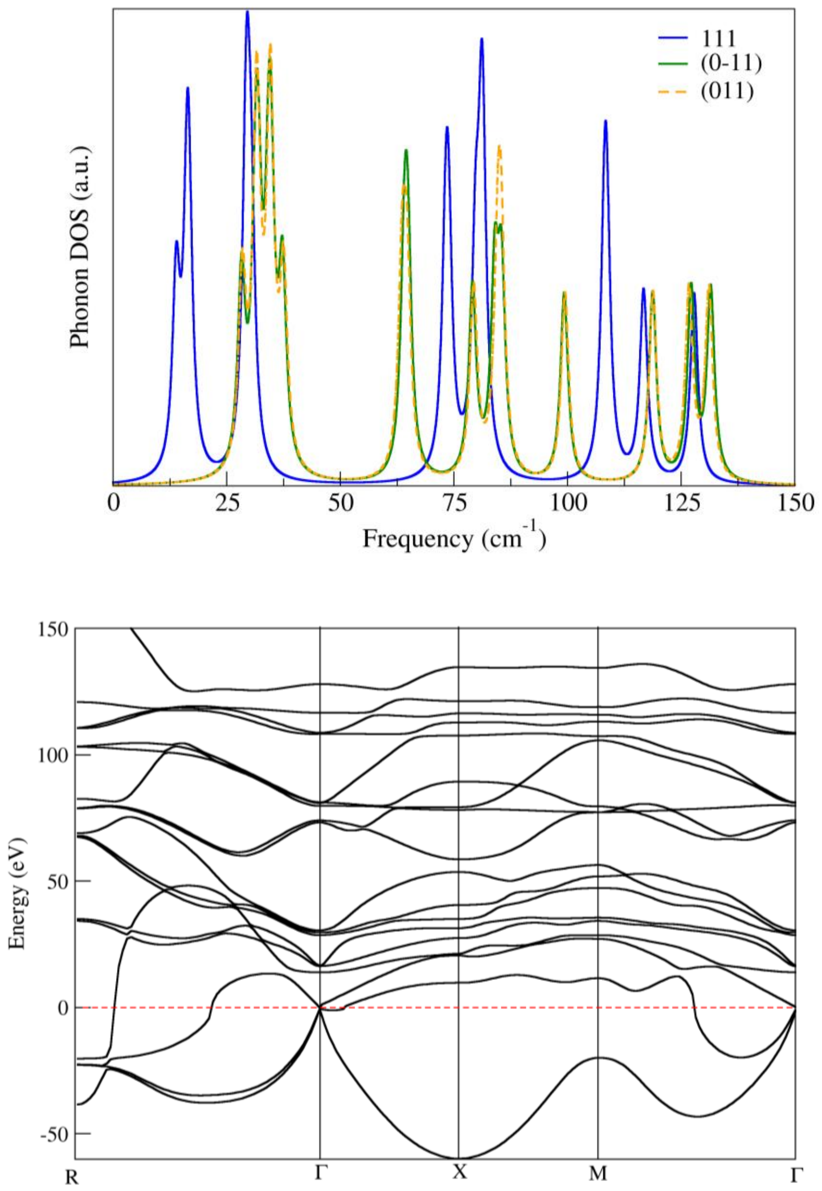

Supplementary Figure 5: Top panel: phonon density of states (DOS) as a function of the energy at the Γ point for the cubic phase of $CH_3NH_3PbI_3$ the molecule is oriented along (111) (blue line), (0-11) (green line) and (011) (orange line). The low-energy region in the phonon density of states of $CH_3NH_3PbI_3$ is shown, calculated at the Γ point with the frozen-phonon approximation by using a 2x2x2 supercell. Focusing on the (111) orientation, the vibrations in the 0-50 cm^{-1} range represent correlated phonons of the inorganic and organic sublattices. The Pb-I cage distortions induce liberations (14-16 cm^{-1}) or translations (30 cm^{-1}) of the MA. The other vibrations showed, with the exception of that at 79 cm^{-1} (which involves vibrations of the Pb-I bonds), are related to liberations (73,81 and 108 cm^{-1}) rotation (116 cm^{-1}) and translation (127 cm^{-1}) of the MA with respect to the quasi-static inorganic cage. In the (011) and (0-11) cases, in general the same considerations apply. In this case, however, the molecule is more constrained in two of the three directions in space, thus some liberations are shifted to lower energies, while others are pushed up because more entangled with the Pb-I vibrations. Lower panel: phonon dispersion of $CH_3NH_3PbI_3$ with (111) oriented molecules. Outside the Γ point some acoustic phonon modes become unstable, showing negative frequencies. This is expected, because the system spontaneously tends to its zero-temperature ground state, where the PbI_6 octahedra assume an alternating tilting and the symmetry is reduced from cubic to orthorhombic unit cell. By visual inspection, we verified that the negative branches induce tilting of the octahedra.

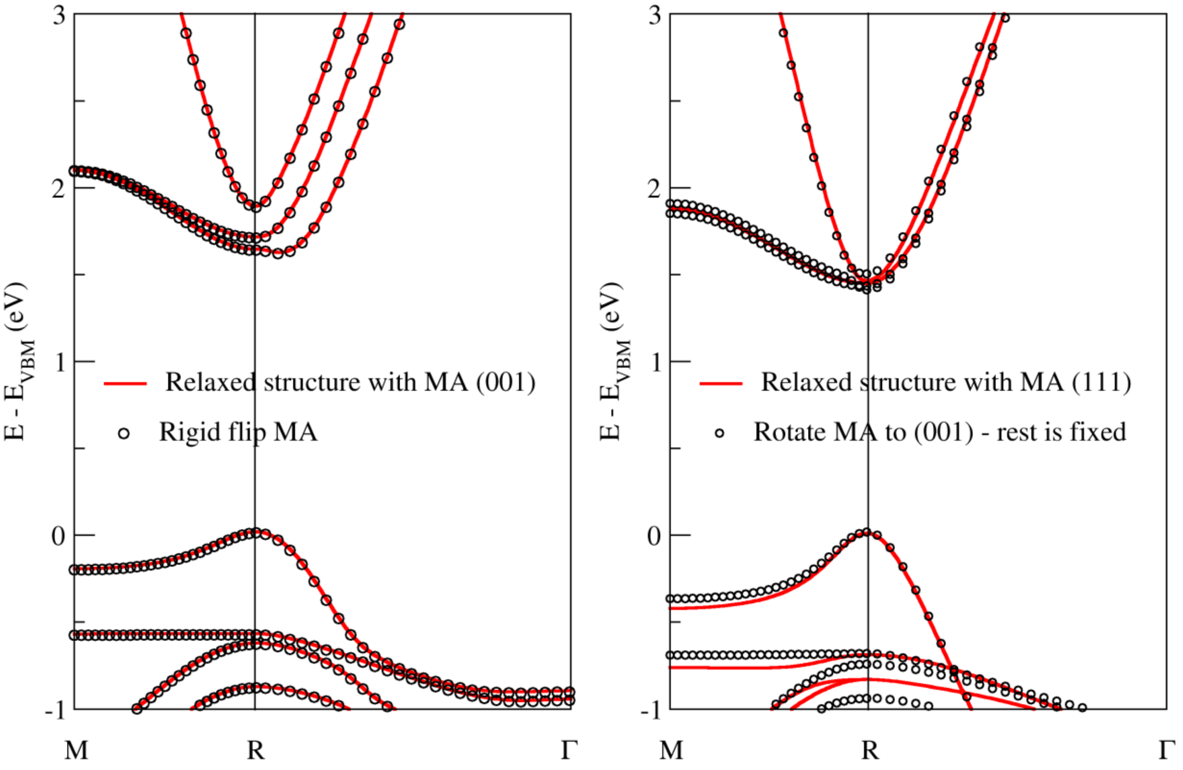

Supplementary Figure 6: Left panel: electronic band structure for the relaxed geometry of $CH_3NH_3PbI_3$ with the MA oriented along (011) (red line) and upon a rigid flip of the MA dipole moment without further optimisation (circles). Right panel: electronic band structure for the relaxed geometry of $CH_3NH_3PbI_3$ with the MA oriented along (111) (red line) and upon a rigid rotation of the MA dipole moment from the (111) to the (011) direction (circles). In both cases, the location of the CBM and VBM remain unaltered by varying the orientation of the MA dipole moment.

	**111 CH_3NH_3 orientation**			**011 CH_3NH_3 orientation**		
a_1 (Å)	6.341616	0.066671	0.066651	6.279065	0.016599	-0.008248
a_2 (Å)	0.066654	6.341654	0.066870	-0.017861	6.376247	-0.139818
a_3 (Å)	0.066796	0.066901	6.341641	0.008476	-0.047483	6.340938
**Atomic Coordinates (Å)**						
Pb	-0.009675	-0.009774	-0.009898	3.136510	3.288526	2.964311
I	3.137045	-0.009240	-0.009361	3.127458	6.488224	2.635877
I	-0.009179	3.136977	-0.009470	6.276499	3.110303	2.846567
I	-0.009200	-0.009368	3.136841	3.140087	3.442878	6.055399
C	3.002872	3.002888	3.002777	6.271232	6.645286	5.701902
N	3.863438	3.863801	3.863760	6.274265	5.999923	7.047665
H	2.376979	2.376942	3.644528	6.276696	4.967810	6.996251
H	3.644978	2.377143	2.377013	5.432817	6.261936	7.601383
H	2.377057	3.644758	2.376826	7.115707	6.265956	7.599492
H	4.489487	3.318871	4.489913	5.372627	6.330016	5.163931
H	3.318285	4.489788	4.489768	7.169411	6.333143	5.161401
H	4.489545	4.489973	3.318913	6.269543	7.730371	5.835903

Supplementary Table 1: Lattice vectors (a_1, a_2, a_3) and atomic coordinates in Å of the relaxed $CH_3NH_3PbI_3$ unit cells using PBE with TS09-van der Waals interaction for differently oriented positions of the CH_3NH_3 molecules.
